# A Network Pharmacology Approach to Reveal the Underlying Mechanisms of Rhizoma Dioscoreae Nipponicae in the Treatment of Asthma

**DOI:** 10.1155/2022/4749613

**Published:** 2022-03-30

**Authors:** Weiyi Wang, Liying Xu, Lingming Zhou, Shanhong Wan, Libin Jiang

**Affiliations:** ^1^Department of Respiratory Medicine, The First Affiliated Hospital of Zhejiang Chinese Medical University (Zhejiang Provincial Hospital of Traditional Chinese Medicine), Hangzhou, Zhejiang, China; ^2^Department of Emergency, The First Affiliated Hospital of Zhejiang Chinese Medical University (Zhejiang Provincial Hospital of Traditional Chinese Medicine), Hangzhou, Zhejiang, China; ^3^Department of Respiratory Medicine, Zhejiang Chinese Medical University, Hangzhou, Zhejiang, China

## Abstract

**Background:**

In this study, network pharmacological methods were used to analyze the targets of Rhizoma Dioscoreae Nipponicae (RDN) and investigate the potential underlying mechanism of RDN in the treatment of asthma.

**Methods:**

Asthma-related targets were obtained from the GeneCards and DisGeNET databases. The bioactive components of RDN were obtained from the Traditional Chinese Medicine Systems Pharmacology Database and Analysis Platform database, and the targets of these compounds were predicted using the BATMAN-TCM database. The network of RDN component targets was constructed using Cytoscape. A protein-protein interaction (PPI) network was constructed in Cytoscape to determine the potential targets of RDN for the treatment of asthma. The hub genes of RDN in the treatment of asthma were screened using network topological parameters. Gene ontology (GO) and the KEGG pathways were analyzed. Molecular docking and in vivo experiments were performed to validate the network pharmacology results.

**Results:**

A total of four bioactive components and 55 targets were identified. The results of the enrichment analysis suggested that the treatment of asthma with RDN involved signaling pathways, such as those related to systemic lupus erythematosus, alcoholism, viral carcinogenesis, the cell cycle, prostate cancer, transcriptional misregulation in cancer, hepatitis B, thyroid hormone signaling, and PI3K-AKT signaling, as well as other signaling pathways. Molecular docking showed that the active components of RDN could stably bind to the predicted target. In vivo experiments showed that RDN could regulate the expression of target genes and inhibit the activation of the PI3K-AKT signaling pathway.

**Conclusion:**

To a certain extent, this study reveals the potential bioactive components and molecular mechanisms of RDN in the treatment of asthma and provides new insights for the development of new drugs for asthma.

## 1. Introduction

Asthma is a common chronic condition characterized by chronic airway inflammation, which leads to increased bronchial hyperresponsiveness and mucus hypersecretion [1]. Asthma can be caused by a variety of stimuli, including viral infections, exposure to allergens and/or pollutants, smoking, sudden temperature variations, stress, and exercise [2, 3]. The global incidence of asthma was 43.12 million cases/year in 2017, with asthma incidence and mortality rates of 272.68 million and 490,000 cases, respectively, in the same year [4]. Asthma has a definite impact on the national economies of countries worldwide [5]. Presently, some patients with asthma cannot be effectively treated with corticosteroids. Therefore, it is necessary to develop new and significant treatments for asthma. Clinical studies have found that traditional Chinese medicine (TCM), a type of alternative medicine, has good clinical effects on asthma [6–8]. As a supplementary medicine, TCM is widely used in the treatment of asthma with good results and few side effects [9].

Rhizoma Dioscoreae Nipponicae (RDN) (ChuanShanlong) is indicated in many medical books and has various physiological effects, such as clearing heat, decreasing phlegm, relieving cough and asthma, dispersing blood stasis, and relieving pain [10]. Several findings have shown that RDN alleviates rheumatoid arthritis, treats hyperuricemia, improves thyroiditis, and relieves pulmonary fibrosis [11–14]. Moreover, our previous studies showed that RDN exhibited therapeutic effects in mice with chronic asthma [15–18]. However, the mechanisms of RDN in asthma treatment remain unclear.

TCM can act on multiple biological processes through multiple targets and bioactive components to treat diseases. Network pharmacology is a branch of pharmacology that is based on systems biology and multiple pharmacology theories and primarily focuses on biomolecular networks [19]. At present, network pharmacology is widely used in TCM research to reveal the mechanisms of complex herbal formulae by discovering bioactive ingredients and biomarkers. Furthermore, molecular docking is an important process in structural molecular biology and in computer-aided drug design for the development of new medicines [20].

Here, we analyzed the role of RDN in asthma treatment using a network pharmacology approach, and the results were validated by molecular docking and in vivo experiments.

## 2. Materials and Methods

### 2.1. Screening and Obtaining the Bioactive Components and Targets of RDN

In this study, the Traditional Chinese Medicine Systems Pharmacology Database and Analysis Platform (TCMSP, https://ibts.hkbu.edu.hk/LSP/tcmsp.php) was used to determine the components of RDN [21]. TCM is mostly administered orally and exerts a variety of biological effects through its action on organs and tissues after absorption, distribution, metabolism, and excretion (ADME) in vivo, known as the pharmacokinetics of TCM. Oral bioavailability (OB) and drug-like (DL) are important parameters of ADME [22–24]. Components with OB ≥ 30% and DL ≥ 0.18 were selected as the main candidate bioactive components. In addition, more active components of RDN with significant pharmacological effects were supplemented based on previous studies [25].

BATMAN-TCM [26] was used to retrieve and predict the targets of the main bioactive components in RDN. In addition, components without potential targets were excluded. The targets were collected with a score cutoff of ≥40. The obtained targets, including the name, gene ID, and organism, were confirmed using the UniProt protein database (https://www.uniprot.org) [27]. The duplicate targets were removed and then uploaded to the Cytoscape platform to construct the herb-component-target network.

### 2.2. Identification of Asthma-Related Targets

The keyword “asthma” was used as the search term to collect disease targets based on the GeneCards (https://www.genecards.org) [28] and DisGeNET (https://www.disgenet.org) [29] databases.

The asthma-related targets obtained from the last search were uploaded to the STRING database (https://string-db.org/) to analyze the relational value and were then imported to the Cytoscape platform to construct the asthma target network.

### 2.3. Construction of the Protein-Protein Interaction (PPI) Network

A PPI network was constructed and visually analyzed using the Cytoscape plugin BisoGenet, which contains six PPI databases: IntAct Molecular Interaction Database (IntAct), Biomolecular Interaction Network Database (BIND), Biological General Repository for Interaction Datasets (BioGRID), Database of Interacting Proteins (DIP), Molecular INTeraction Database (MINT), and Human Protein Reference Database (HPRD) [30]. PPI networks of RDN targets and asthma-related targets were constructed using Cytoscape.

Central network evaluation is considered a primary method for screening core proteins in PPI networks. First, the PPI networks of RDN targets and asthma-related targets were merged into an intersection. Next, the Cytoscape plugin CytoNCA was applied to assess the intersection from the six centrality measures: betweenness centrality (BC), closeness centrality (CC), degree centrality (DC), eigenvector centrality (EC), network centrality (NC), and the local average connectivity-based method (LAC) [31]. In addition, CytoHubba, a Cytoscape plugin, was used to select the hub genes [32].

### 2.4. GO and KEGG Pathway Enrichment Analysis

Gene Ontology (GO) and Kyoto Encyclopedia of Genes and Genomes (KEGG) pathway enrichment analyses were carried out using the DAVID Bioinformatics Resources 6.8 database. GO analysis included three aspects: molecular function (MF), cellular component (CC), and biological process (BP).

### 2.5. Chemical Analysis of RDN

The RDN test sample was prepared according to a previous study [33]. Standard diosgenin was purchased from the Tauto Biological Technology Company (China). The chemical constituents of RDN were determined using an Agilent 1260 high-performance liquid chromatography (HPLC) system (Agilent, CA, USA). Samples (10 *μ*L) were separated using an Agilent ZORBAX Eclipse Plus C18 column (4.6 mm × 250 mm, 5 *μ*m) at 30°C, with mobile phases consisting of water (A) and acetonitrile (B). The following gradient conditions were used: 0–5 min, 15% B; 5–30 min, 15–40% B; and 30–50 min, 40–50% B. The flow rate was 1.0 mL/min, and the detection wavelength was 203 nm. Chromatograms are shown in Supplementary Figure 1. The retention time of diosgenin was 7.439 min.

### 2.6. Molecular Docking Analysis

The chemical structures of the RDN ingredients were downloaded from the ZINC website (https://zinc15.docking.org) [34], and three-dimensional structures of the hub genes were downloaded from the RCSB Protein Data Bank (PDB, https://www.pdb.org/) [35]. AutoDockTools (v1.5.6) and PyMol (v2.3.0) were used to remove water molecules, add polar hydrogen, and redistribute charge. Molecular docking was performed using AutoDock Vina (v1.1.2) and visualized using PyMol (v2.3.0).

### 2.7. Asthma Model Establishment and Treatment

Female BALB/c mice (6–8 weeks old, Shanghai SIPPR-BK Laboratory Animal Co. Ltd., China) were housed in a specific pathogen-free environment with a 12-h light-dark cycle, temperature of 23 ± 2°C, and had free access to water and food. All animal experiments were approved by the Ethics Committee of Animal Experiments of the First Affiliated Hospital of Zhejiang Chinese Medical University.

After one week, mice were randomly divided into six groups (*n* = 6): control group, model group, model + RDN group, model + AKT agonist group, model + AKT agonist + RDN group, and model + prednisone acetate (PA) group. Mice were challenged by intraperitoneal injection of 0.2 ml of saline solution containing 50 *μ*g of ovalbumin (grade V, Sigma-Aldrich, MO, USA) and 0.8 mg of aluminum hydroxide (Sigma-Aldrich) on days 0–3 and day 14. On day 14, day 17, day 20, day 23, and day 27, mice in the control group received inhaled phosphate-buffered saline using an ultrasonic nebulizer (402AI, Yuyue Medical Equipment Co. Ltd, China) for 20 min each time, and the rest of the mice received inhaled 2% ovalbumin solution. On days 14–27, mice in the model, model + RDN, model + AKT agonist (SC79, Sigma-Aldrich), model + AKT agonist + RDN, and model + PA (Sigma-Aldrich) groups were challenged with ovalbumin and were administered RDN (5 mg/kg administered by gavage), AKT agonist (10 mg/kg intraperitoneally), PA (5 mg/kg administered by gavage), or AKT agonist + RDN. Mice in the control group were challenged with phosphate-buffered saline.

### 2.8. Hematoxylin and Eosin (H&E) Staining

The lung tissues were collected and infused with 4% paraformaldehyde and were then dehydrated, paraffin-embedded, and cut into 5-*µ*m-thick sections. The sections were then stained with hematoxylin for 3 min and eosin for 3 min. Finally, the pathological morphology of the mouse lung tissue was observed using a digital camera (Leica Microsystems Inc., IL, USA).

### 2.9. Quantitative Real-Time PCR (qRT-PCR) Analysis

Total RNA was extracted from lung tissues using TRIzol® Reagent (Invitrogen, CA, USA) and reverse transcribed into cDNA using the PrimeScript™ RT Reagent Kit (Takara, Japan). qRT-PCR was performed on a 7500 Real-Time PCR System (ThermoFisher Scientific, MD, USA) at 95°C for 3 min, 40 cycles of 95°C for 12 s, and 62°C for 40 s. The primers used are listed in Supplementary Table 1. The relative expression of genes was calculated using the 2^−ΔΔCT^ method, and GAPDH was used as an internal control.

### 2.10. Western Blotting

Total proteins were extracted using RIPA lysis buffer (Beyotime, China), separated by 10% sodium dodecyl sulfate-polyacrylamide gel electrophoresis, and transferred to polyvinylidene fluoride membranes. The membranes were blocked with 5% skim milk for 1 h and incubated with the primary antibodies (1 : 1000, Abcam Co., MA, USA) at 4°C overnight and then incubated with the secondary antibodies at room temperature for 1 h. Finally, enhanced chemiluminescent reagents (FDbio, China) were used to visualize the protein bands.

### 2.11. Statistical Analysis

Data are presented as the means with standard deviation and were analyzed using SPSS 27.0. The differences between the groups were analyzed by one-way ANOVA, followed by Tukey's test. Differences were considered statistically significant at *p* < 0.05.

## 3. Results

### 3.1. The Compound-Target Network of RDN

**Figure 1:**
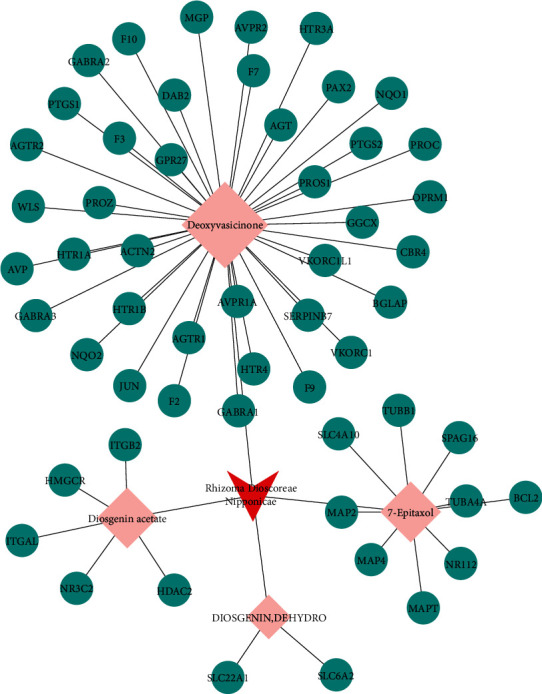
Component-target network of Rhizoma Dioscoreae Nipponicae. The red node represents the herbs, the pink represents the components, and the green represents the targets.

Two components of RDN were obtained from the TCMSP database. Studies have shown that 7-epitaxol and deoxyvasicinone are the major bioactive and principal toxic compounds of RDN. Therefore, 7-epitaxol and deoxyvasicinone were considered as the candidate components in this study. Collectively, we identified a total of 4 bioactive compounds and 55 targets for RDN, the details of which are shown in Tables 1 and 2. We subsequently constructed a compound-target network of RDN using Cytoscape (Figure 1).

### 3.2. GO and KEGG Pathway Enrichment Analyses of RDN

**Figure 2:**
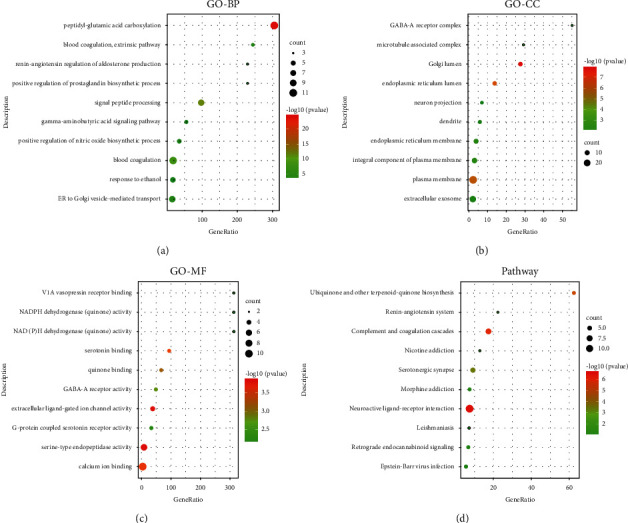
Gene ontology and KEGG pathway enrichment analysis of the bioactive compounds of Rhizoma Dioscoreae Nipponicae. (a) Representative bubble plots of the biological processes (BP) of the identified targets. (b) Representative bubble plots of the cellular components (CC) of the identified targets. (c) Representative bubble plots of the molecular function (MF) of the identified targets. (d) Representative bubble plots of the KEGG pathway of the identified targets. Gene ratio = count/set size.

To further investigate the 55 targets of RDN, GO enrichment analysis was performed using GO terms from the BP, CC, and MF categories. The resultant targets of RDN were classified into 130 BP terms, 27 CC terms, and 31 MF terms, according to *p* < 0.01. The GO analysis results with the top 10 markedly enriched BP, CC, and MF terms are shown in Figures 2(a)–2(c). KEGG pathway enrichment analysis showed that the targets of the components in RDN were enriched in 12 associated pathways (*p* < 0.01). The top 10 KEGG pathways in which the targets of RDN were enriched are presented in Figures 2(d) and Table 3.

### 3.3. The Network of Asthma-Related Targets

The occurrence and development of asthma involve the co-regulation of multiple genes. Investigation of gene and gene-environment interactions is beneficial for elucidating the pathogenesis of asthma. In this study, 55 asthma-related targets were obtained from human genomic databases. The number of these targets in the GeneCards and DisGeNET databases was 19 and 38, respectively. These targets are detailed in Table 4.

### 3.4. The PPI Network of RDN and Asthma

**Figure 3:**
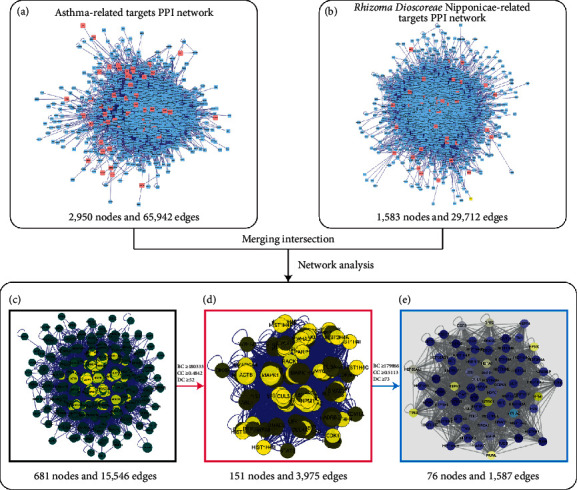
Identification of the core targets for Rhizoma Dioscoreae Nipponicae against asthma. (A) The PPI network of Rhizoma Dioscoreae Nipponicae-related targets (2,950 nodes and 65,942 edges). (B) The PPI network of asthma-related targets (1,583 nodes and 29,712 edges). (C) Intersection of PPI networks (681 nodes and 15,546 edges). (D) PPI network obtained with the screening criteria of BC ≥ 180333, CC ≥ 0.4842, and DC ≥ 52 (151 nodes and 3,975 edges). (E) Core-target PPI network obtained with the screening criteria of BC ≥ 179866, CC ≥ 0.5113, and DC ≥ 73 (76 nodes and 1,587 edges). Abbreviations: BC, betweenness centrality; CC, closeness centrality; DC, degree centrality.

The PPI networks of RDN and asthma were constructed using BisoGenet. The asthma-related target network was constructed with 1,583 nodes and 29,712 edges (Figure 3(A)). In addition, the PPI network of RDN-related genes was constructed with 2,950 nodes and 65,942 edges (Figure 3(B)). These two networks were subsequently merged into an intersection with 681 nodes and 15,546 edges (Figure 3(C)). CytoNCA was used to assess the intersection of the PPI network by topological analysis. An RDN on asthma PPI network, with 151 nodes and 3,975 edges, was first screened based on the criteria of “BC ≥ 180333, CC ≥ 0.4842, and DC ≥ 52” (Figure 3(D)). A core-target PPI network was further screened using the criteria “BC ≥ 179866, CC ≥ 0.5113, and DC ≥ 7,” and consisted of 76 nodes and 1,587 edges (Figure 3(E)).

### 3.5. GO Functional Enrichment and Pathway Analysis

GO annotation showed that the targets of the PPI network were classified into 191 BP terms, 49 CC terms, and 68MF terms according to *p* < 0.01. KEGG pathway analysis revealed that the targets of the PPI network were assigned to 43 KEGG pathways based on *p* < 0.01.

**Figure 4:**
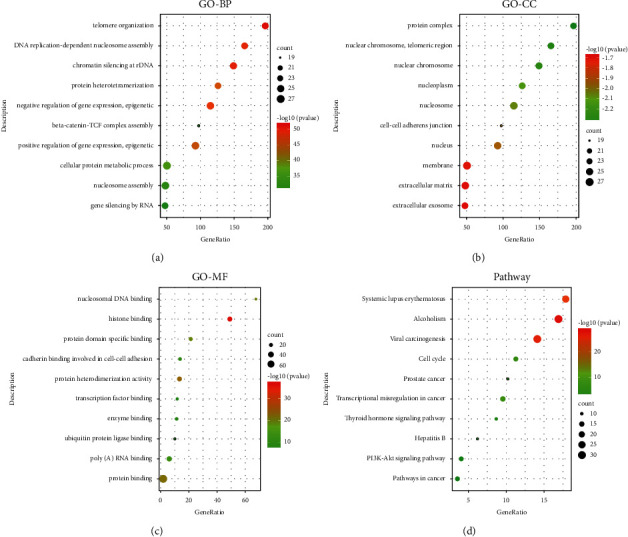
Gene ontology and KEGG pathway enrichment analysis of the core targets for Rhizoma Dioscoreae Nipponicae against asthma. (a) Representative bubble plots of the biological processes (BP) of the core targets. (b) Representative bubble plots of the cellular components (CC) of the core targets. (c) Representative bubble plots of the molecular function (MF) of the core targets. (d) Representative bubble plots of the KEGG pathway of the core targets. Gene ratio = count/set size.

In the BP ontology, the targets of the PPI network were primarily associated with telomere organization, DNA replication-dependent nucleosome assembly, chromatin silencing at rDNA, protein heterotetramerization, negative regulation of gene expression, epigenetics, beta-catenin-TCF complex assembly, positive regulation of gene expression, cellular protein metabolic processes, nucleosome assembly, and gene silencing by RNA (Figure 4(a)).

For the CC ontology, the targets were located mainly in the protein complex, nuclear chromosome, telomeric region, nucleoplasm, nucleosome, cell-cell adherens junction, nucleus, membrane, extracellular matrix, and extracellular exosome (Figure 4(b)).

Based on GO annotation of MF, it was observed that the targets were mainly involved in nucleosomal DNA binding, histone binding, protein domain-specific binding, cadherin binding involved in cell-cell adhesion, protein heterodimerization activity, transcription factor binding, enzyme binding, ubiquitin-protein ligase binding, poly(A) RNA binding, and protein binding (Figure 4(c)).

**Figure 5:**
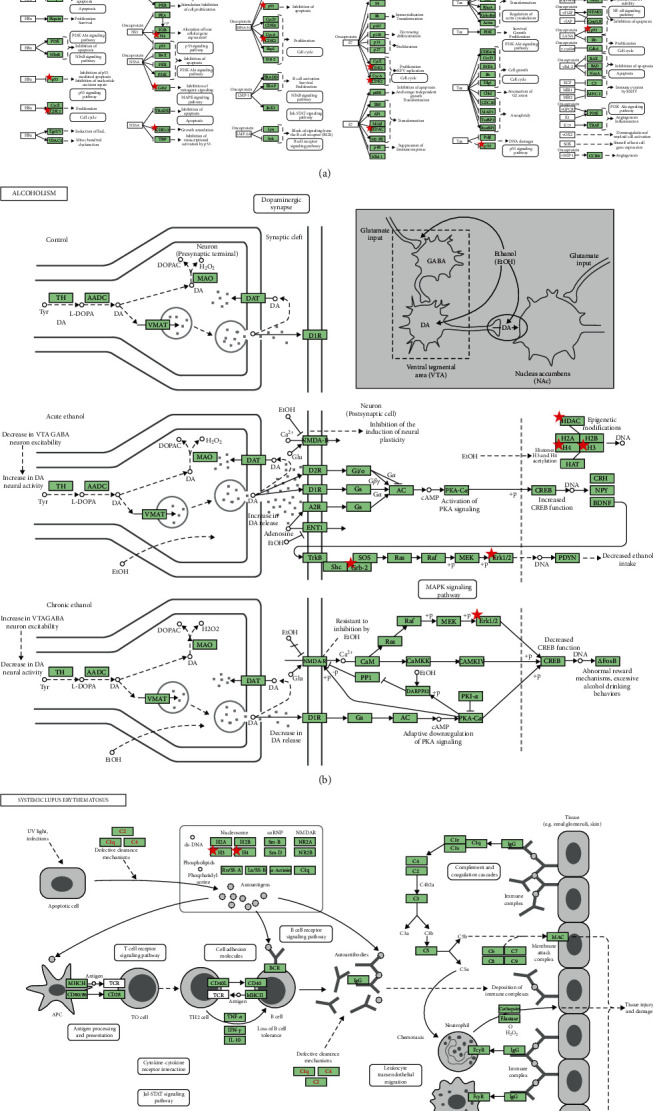
Distribution of key targets in the most enriched pathways. (a) Distribution of key targets in viral carcinogenesis. (b) Distribution of key targets in alcoholism. (c) Distribution of key targets in systemic lupus erythematosus. The green rectangle represents the key targets.

In addition to functional GO annotation, KEGG pathway analysis was also conducted on the targets of the PPI network. As shown in Figure 4(d), KEGG pathway enrichment analysis suggested that the targets were mainly related to signaling pathways, such as systemic lupus erythematosus, alcoholism, viral carcinogenesis, cell cycle, prostate cancer, transcriptional misregulation in cancer, the thyroid hormone signaling pathway, hepatitis B, the PI3K-Akt signaling pathway, and pathways in cancer. The distribution of the key targets in systemic lupus erythematosus, alcoholism, and viral carcinogenesis pathways is shown in Figure 5. The results indicated that the action targets of the main bioactive components of RDN were distributed in different metabolic pathways. The “multicomponents, multitargets, and multi-pathways” mutual regulation is a possible mechanism in the treatment of asthma.

### 3.6. Enrichment Analysis of the Hub Genes

**Figure 6:**
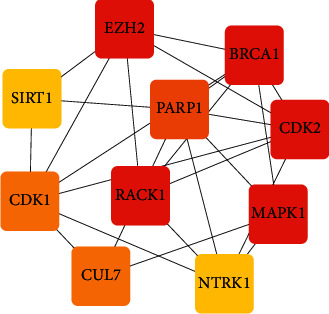
The top 10 hub genes network of Rhizoma Dioscoreae Nipponicae against asthma.

Based on the core-target PPI network, hub genes were selected using CytoHubba. The results showed that EZH2, SIRT1, CDK1, CUL7, NTRK1, MAPK1, RACK1, PARP1, CDK2, and BRCA1 were the top 10 hub nodes in the core-target PPI network. The PPI network of the hub genes is shown in Figure 6.

### 3.7. Molecular Docking Verification

**Figure 7:**
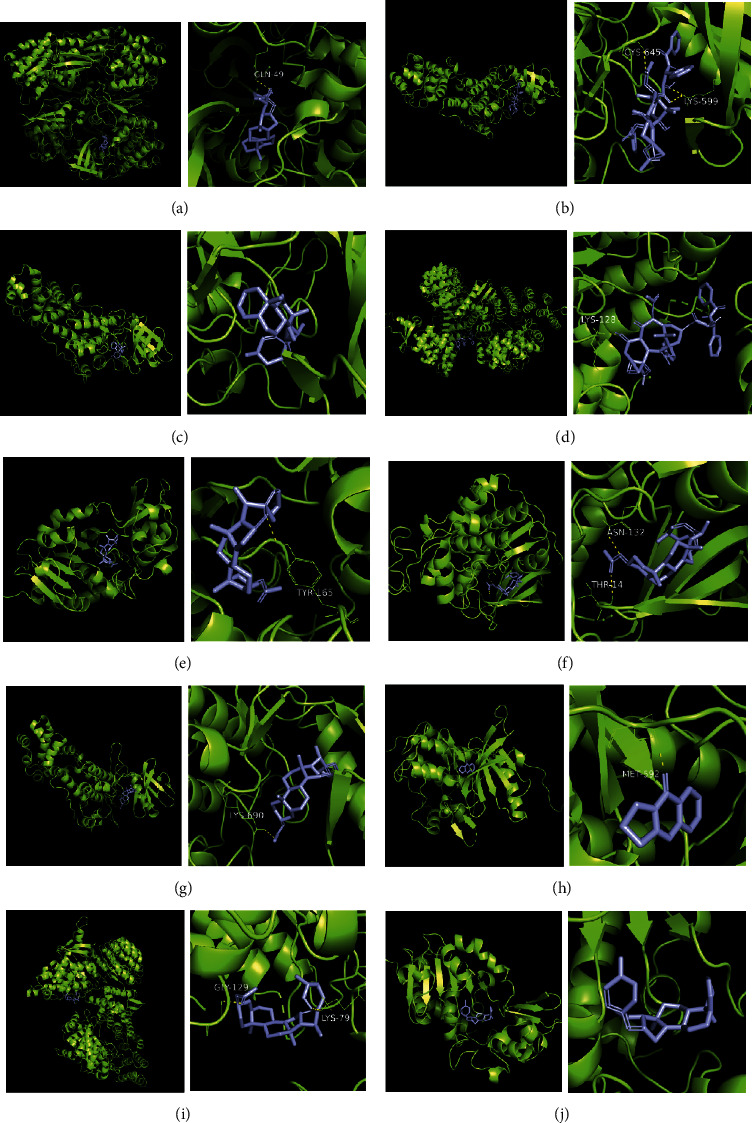
Molecular docking of bioactive compound hub genes. (a) Diosgenin, dehydro to CDK1, affinity = −10.2 kcal/mol. (b) 7-Epitaxol to RACK1, affinity = −9.3 kcal/mol. (c) Diosgenin, dehydro to RACK1, affinity = −9.3 kcal/mol. (d) 7-Epitaxol to CUL7, affinity = −9.1 kcal/mol. (e) Diosgenin acetate to SIRT1, affinity = −9.1 kcal/mol. (f) Diosgenin acetate to CDK2, affinity = −9 kcal/mol. (g) Diosgenin acetate to RACK1, affinity = −8.5 kcal/mol. (h) Deoxyvasicinone to NTRK1, affinity = −8.4 kcal/mol. (i) Diosgenin acetate to CUL7, affinity = −8.4 kcal/mol. (j) Diosgenin, dehydro to SIRT1, affinity = −8.3 kcal/mol.

The molecular docking assay was used to verify the binding ability of the bioactive components of RDN (detailed information regarding the bioactive components is listed in Table 5) and the top nine hub genes (CDK2, PDB: 1b39; SIRT1, PDB: 4jt8; RACK1, PDB: 4u6r; EZH2, PDB: 5ij8; NTRK1, PDB: 5wr7; MAPK1, PDB: 6g54; CDK1, PDB: 6gu7; CUL7, PDB: 6o60; and PARP1, PDB: 7kkq). As shown in Table 6, we obtained 32 pairs of docking results when setting the threshold affinity to < −5 kcal/mol. Among them, diosgenin, dehydro had a strong binding ability with CDK1 (affinity = −10.2 kcal/mol), RACK1 (affinity = −9.3 kcal/mol), and SIRT1 (affinity = −8.3 kcal/mol). Additionally, 7-epitaxol had a strong binding ability with RACK1 (affinity = −9.3 kcal/mol) and CUL7 (affinity = −9.1 kcal/mol). Diosgenin acetate had a strong binding ability with SIRT1 (affinity = −9.1 kcal/mol), CDK2 (affinity = −9 kcal/mol), RACK1 (affinity = −8.5 kcal/mol), and CUL7 (affinity = −8.4 kcal/mol). Deoxyvasicinone had a strong binding ability with NTRK1 (affinity = −8.4 kcal/mol) (Figure 7).

### 3.8. Verification with Animal Experimentation

**Figure 8:**
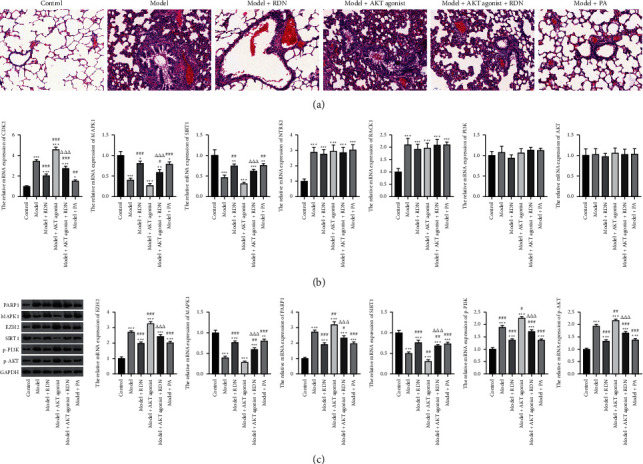
Action of Rhizoma Dioscoreae Nipponicae against asthma via the PI3K-AKT pathway. (a) Hematoxylin and eosin staining of the lung slices in each group. (b) The expression of SIRT1, MAPK1, CDK2, RACK1, NTRK1, PI3K, and AKT in lung tissues from asthma mice was determined by qRT-PCR. (c) Western blot was used to detect the expression of PARP1, EZH2, MAPK1, SIRT1, p-PI3K, and p-AKT. ^*∗*^*p* < 0.05, ^*∗∗*^*p* < 0.01, and ^*∗∗∗*^*p* < 0.001 vs. control group; ^#^*p* < 0.05, ^##^*p* < 0.01, and ^###^*p* < 0.001 vs. model group; ^ΔΔΔ^*p* < 0.001 vs. model + AKT agonist group.

To further verify the therapeutic effect of RDN on asthma, we created a mouse model of ovalbumin-induced asthma. H&E staining results showed that the alveoli of mice in the control group had clear texture, no obvious inflammatory cell infiltration, regular bronchial lumen, no obvious thickening, intact airway mucosal epithelial cells, and an intact alveolar wall structure. The lung tissues of mice in the model and model + AKT agonist groups showed clear alveolar texture, a small amount of inflammatory cell infiltration, regular bronchial lumen, and an intact airway mucosal epithelium; the damage to the lung tissue in the model + AKT agonist + RDN group was significantly reduced compared with that observed in the model group, and the inflammatory cell infiltration around the bronchi was reduced, but still heavier than that observed in the model + PA and model + RDN groups (Figure 8(a)).

We also detected the expression levels of the predicted targets and the PI3K-AKT pathway using qRT-PCR and Western blot analysis. As shown in Figure 8(b), compared with the control group, the expression of SIRT1 and MAPK1 was significantly decreased, while CDK2 expression significantly increased in the model and model + AKT agonist groups. This phenomenon was reversed by RDN administration. In addition, the expression of RACK1 and NTRK1 was significantly upregulated in the other groups compared with that in the control group, with no significant differences between the other groups. Furthermore, PI3K and AKT expression was not significantly different between the groups.

Western blotting showed that the expression of PARP1, EZH2, p-PI3K, and p-AKT was significantly increased in the model and model + AKT agonist groups compared with that in the control group, in contrast to the significantly decreased expression of MAPK1 and SIRT1. However, this inhibition or enhancement was reversed by the administration of RDN (Figure 8(c)).

## 4. Discussion

Asthma is a common, complex respiratory disease with high morbidity and mortality rates. RDN has been demonstrated to have an effect on asthma, but its pharmacological mechanisms have not been elucidated systematically and comprehensively. In this study, through network pharmacology, we further explored the mechanisms of RDN in asthma. Three aspects of our observations gained our attention. First, four bioactive compounds of RDN were identified. Second, systemic lupus erythematosus, alcoholism, viral carcinogenesis, cell cycle, and prostate cancer were considered as the key sections of RDN in asthma treatment at the gene level. Third, ten hub targets for asthma were enriched in pathological changes in airway inflammation and remodeling as well as other diseases.

In this study, we analyzed the targets of RDN and identified potential signaling pathways. There were a total of four bioactive components in the RDN with the standard. The main bioactive components of RDN are 7-epitaxol and deoxyvasicinone. The bioactive component-target network showed that these compounds had multiple targets. 7-Epitaxol (OB = 45.18, DL = 0.24) is a metabolite of taxol and has the same activity as taxol [36, 37]. Taxol has been reported to have a good clinical effect on asthma [38, 39]. Deoxyvasicinone (OB = 56.29, DL = 0.1) consists of a quinazolinone moiety conjugated with pyrrolidine, a naturally occurring alkaloid with antibacterial, anti-inflammatory, and antiproliferative activities [40, 41]. Moreover, HLPC analysis showed that diosgenin is an important component of RDN.

The results of GO enrichment analysis showed that all targets in the PPI network were mainly involved in beta-catenin-TCF complex assembly and other biological processes. An increasing body of evidence suggests that beta-catenin-TCF plays a key role in asthma. Beta-catenin-TCF is a pleiotropic cytokine involved in airway inflammatory responses and fibrotic tissue remodeling in the pathogenesis of asthma [42, 43].

KEGG pathway enrichment analysis showed enrichment in systemic lupus erythematosus, alcoholism, the PI3K-AKT signaling pathway, and other signaling pathways. A link between asthma and systemic lupus erythematosus (SLE) has been reported [44]. Charoenngam et al. found a significant association between asthma and an increased risk of SLE using a meta-analysis technique [45]. Collectively, alcoholism induces airway mast cells to release histamine, which exacerbates asthma in susceptible populations [46]. The PI3K-Akt pathway is thought to be closely associated with airway inflammation and is regulated by various upstream proteins. Azithromycin has been reported to affect airway remodeling in asthma via the PI3K/Akt pathway [47]. Brg1 exacerbates asthma airway inflammation by inhibiting the PI3K/Akt/mTOR pathway [48]. In addition, lncRNA-CASC7 enhances corticosteroid sensitivity by inhibiting the PI3K/AKT signaling pathway by targeting miR-21 in asthma [49].

In this study, 10 hub targets of RDN were identified for asthma treatment, and RACK1 and SIRT1 were shown to play important roles in asthma. The receptor for activated protein kinase C1 (RACK1), a component of the ribosome, located in the basal airway epithelium [50], plays an important role in a variety of biological responses, including cell growth, differentiation, and migration. Apoptosis and epithelial-mesenchymal transition (EMT) are two key components in the pathogenesis of asthma, both of which are mediated by TGF-*β*1. Knockdown of RACK1 significantly inhibited apoptosis and reduced TGF-*β*1 upregulation of EMT-related protein levels (N-calmodulin and Snail) in vitro [51]. Sirtuin 1 (SIRT1) is a class III histone deacetylase that plays an important role in the regulation of many pathophysiological processes, such as inflammation, autoimmunity, and apoptosis [52]. Lai et al. found that myeloid-specific SIRT1 deficiency exacerbated the airway inflammatory response in a mouse model of allergic asthma [53], which also suggests an important role for SIRT1 gene activation in asthma treatment.

Finally, we validated the results of the network pharmacological approach using molecular docking and in vivo experiments in asthmatic mice. The molecular docking assay showed that the binding energy of the active compounds in RDN to the predicted targets was less than −5 kcal/mol, suggesting that RDN binds well to the therapeutic targets in asthma. In addition, Western blot results showed that RDN reduced the expression of p-PI3K and p-AKT, suggesting that RDN resistance to asthma may be achieved through the PI3K-AKT pathway.

## 5. Conclusions

In summary, network pharmacology showed that the bioactive components of RDN, particularly 7-epitaxol and deoxyvasicinone, could act on multiple targets. RDN was effective in treating asthma mainly through the pathways related to systemic lupus erythematosus and alcoholism, the PI3K-AKT signaling pathway, and other signaling pathways. The results of molecular docking, qRT-PCR, and Western blot analysis suggest that PARP1, CDK2, and MAPK1 might play important roles in the treatment of asthma. This study provides a basis for the treatment of asthma by RDN from the perspective of network pharmacology. These findings have guiding significance for the clinical application of RDN and provide new clues for the future study and development of new drugs. Of course, our study also needs more *in vitro* and *in vivo* experiments to verify the proposed molecular targets.

## Figures and Tables

**Table 1:** Bioactive compounds of *Rhizoma Dioscoreae* Nipponicae.

Chemical	Molecular formula	Molecular weight (g/mol)	OB (%)	DL	Compound 2D structure
7-Epitaxol	C_47_H_51_NO_14_	853.99	45.18	0.24	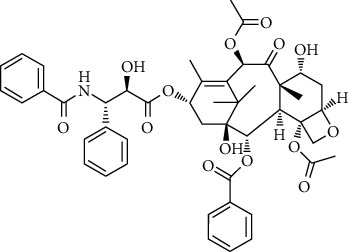
Deoxyvasicinone	C_11_H_10_N_20_	186.21	56.29	0.1	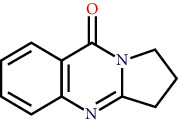
Diosgenin acetate	C_29_H_44_O_4_	456.7	16.72	0.72	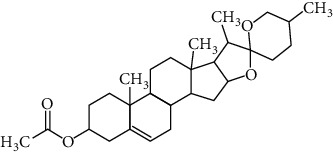
Diosgenin, dehydro	C_27_H_40_O_2_	396.6	12.67	0.82	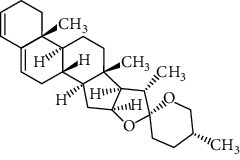

**Table 2:** Targets of *Rhizoma Dioscoreae* Nipponicae.

Gene symbol	Protein name
HTR4	5-hydroxytryptamine receptor 4
GABRA2	Gamma-aminobutyric acid receptor subunit alpha-2
PROS1	Vitamin K-dependent protein S
PROZ	Vitamin K-dependent protein Z
AVPR1A	Vasopressin V1a receptor
NQO2	Ribosyldihydronicotinamide dehydrogenase
HTR1B	5-hydroxytryptamine receptor 1B
GABRA3	Gamma-aminobutyric acid receptor subunit alpha-3
F7	Coagulation factor VII
F9	Coagulation factor IX
GGCX	Vitamin K-dependent gamma-carboxylase
F10	Coagulation factor X
VKORC1	Vitamin K epoxide reductase complex subunit 1
JUN	Transcription factor AP-1
PROC	Vitamin K-dependent protein C
OPRM1	Mu-type opioid receptor
AGTR2	Type 2 angiotensin II receptor
BGLAP	Osteocalcin
PTGS1	Prostaglandin G/H synthase 1
AVPR2	Vasopressin V2 receptor
HTR1A	5-hydroxytryptamine receptor 1A
PTGS2	Prostaglandin G/H synthase 2
HTR3A	5-hydroxytryptamine receptor 3A
GABRA1	Gamma-aminobutyric acid receptor subunit alpha-1
NQO1	NAD(P)H dehydrogenase [quinone] 1
AGTR1	Type 1 angiotensin II receptor
VKORC1L1	Vitamin K epoxide reductase complex subunit 1-like protein 1
F2	Prothrombin
AGT	Alanine-glyoxylate aminotransferase
CBR4	Carbonyl reductase family member 4
AVP	Pyrophosphate-energized vacuolar membrane proton pump 1
PAX2	Paired box protein Pax-2
DAB2	Disabled homolog 2-interacting protein
MGP	Matrix Gla protein
F3	Coagulation factor III
WLS	Protein wntless homolog
GPR27	Probable G protein-coupled receptor 27
SERPINB7	Serpin B7
ACTN2	Alpha-actinin-2
MAP2	Microtubule-associated protein 2
BCL2	Apoptosis regulator Bcl-2
NR1I2	Nuclear receptor subfamily 1 group I member 2
MAPT	Microtubule-associated protein tau
TUBB1	Tubulin beta-1 chain
MAP4	Microtubule-associated protein 4
TUBA4A	Tubulin alpha-4A chain
SPAG16	Sperm-associated antigen 16 protein
SLC4A10	Sodium-driven chloride bicarbonate exchanger
SLC6A2	Sodium-dependent noradrenaline transporter
SLC22A1	Solute carrier family 22 member 1
HMGCR	3-hydroxy-3-methylglutaryl-coenzyme A reductase
ITGB2	Integrin beta-2
ITGAL	Integrin alpha-L
HDAC2	Histone deacetylase 2
NR3C2	Mineralocorticoid receptor

**Table 3:** Top 10 KEGG pathways of *Rhizoma Dioscoreae* Nipponicae.

ID	Description	p.adjust	Count
hsa04080	Neuroactive ligand-receptor interaction	2.27*E* − 07	12
hsa04610	Complement and coagulation cascades	2.05*E* − 06	7
hsa00130	Ubiquinone and other terpenoid-quinone biosynthesis	2.69*E* − 05	4
hsa04726	Serotonergic synapse	3.72*E* − 04	6
hsa04614	Renin-angiotensin system	0.007350602	3
hsa05032	Morphine addiction	0.014516067	4
hsa04723	Retrograde endocannabinoid signaling	0.019155926	4
hsa05033	Nicotine addiction	0.021332245	3
hsa05169	Epstein–Barr virus infection	0.031292166	4
hsa05140	Leishmaniasis	0.060926194	3

**Table 4:** Targets of asthma.

Gene symbol	Gene description
SYK	Tyrosine-protein kinase SYK
MAPK14	Mitogen-activated protein kinase 14
AIMP2	Aminoacyl tRNA synthase complex-interacting multifunctional protein 2
SERPINE1	Plasminogen activator inhibitor 1
IL13	Interleukin-13 receptor subunit alpha-2
POLDIP2	Polymerase delta-interacting protein 2
RAG1	V(D)J recombination-activating protein 1
COL9A3	Collagen alpha-3(IX) chain
COL9A1	Collagen alpha-1(IX) chain
CTNNB1	Catenin beta-1
SCN8A	Sodium channel protein type 8 subunit alpha
MAPK1	Mitogen-activated protein kinase 1
TGFB2	Transforming growth factor beta-2 proprotein
CRK	Adapter molecule crk
ACTB	Actin, cytoplasmic 1
ANXA2	Annexin A2
LGALS9	Galectin-9
COMP	Cartilage oligomeric matrix protein
CYSLTR1	Cysteinyl leukotriene receptor 1
MMP9	Matrix metalloproteinase-9
COL9A2	Collagen alpha-2(IX) chain
EGFR	Epidermal growth factor receptor
GRAP2	GRB2-related adapter protein 2
SEMA7A	Semaphorin-7A
MPPE1	Metallophosphoesterase 1
AHSA1	Activator of 90 kDa heat shock protein ATPase homolog 1
TLR6	Toll-like receptor 6
RNF19A	E3 ubiquitin-protein ligase RNF19A
TRPV4	Transient receptor potential cation channel subfamily V member 4
PLF	Pulmonary function
SSRP1	FACT complex subunit SSRP1
LCN2	Neutrophil gelatinase-associated lipocalin
TRPV1	Transient receptor potential cation channel subfamily V member 1
PDCD5	Programmed cell death protein 5
HMGB1	High mobility group protein B1
TACR1	Substance-P receptor
ALOX5	Polyunsaturated fatty acid 5-lipoxygenase
CCL11	Eotaxin
TNF	Tumor necrosis factor
ADRB2	Beta-2 adrenergic receptor
MUC7	Mucin-7
HNMT	Histamine N-methyltransferase
SCGB3A2	Secretoglobin family 3A member 2
PLA2G7	Platelet-activating factor acetylhydrolase
BRCA2	Breast cancer type 2 susceptibility protein
MIR126	miRNA-126
MIR148A	miRNA-148A
MIR148B	miRNA-148B
MIR152	miRNA-152
HLA-G	HLA class I histocompatibility antigen
ASRT3	Asthma-related traits, susceptibility to, 3
ASRT4	Asthma-related traits, susceptibility to, 4
ASRT8	Asthma-related traits, susceptibility to, 8
ASRT6	Asthma-related traits, susceptibility to, 6

**Table 5:** Chemical structure of active compounds.

Synonyms	CAS	Molecular formula	2D structure
7-Epitaxol	105454-04-4	C_47_H_51_NO_14_	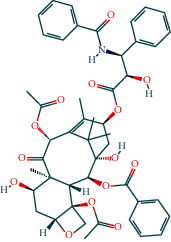
Deoxyvasicinone	530-53-0	C_11_H_10_N_20_	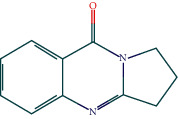
Diosgenin acetate	1061-54-7	C_29_H_44_O_4_	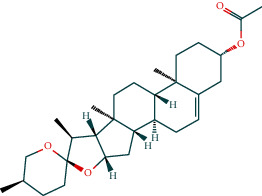
Diosgenin, dehydro	1672-65-7	C_27_H_40_O_2_	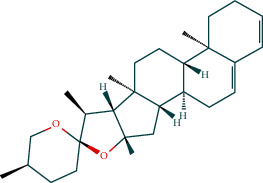

**Table 6:** Results of molecular docking.

Chemical	PDB	Gene	Best affinity
Diosgenin, dehydro	6gu7	CDK1	−10.2
7-Epitaxol	4u6r	RACK1	−9.3
Diosgenin, dehydro	4u6r	RACK1	−9.3
7-Epitaxol	6o60	CUL7	−9.1
Diosgenin acetate	4jt8	SIRT1	−9.1
Diosgenin acetate	1b39	CDK2	−9
Diosgenin acetate	4u6r	RACK1	−8.5
Deoxyvasicinone	5wr7	NTRK1	−8.4
Diosgenin acetate	6o60	CUL7	−8.4
Diosgenin, dehydro	4jt8	SIRT1	−8.3
Diosgenin, dehydro	1b39	CDK2	−8.3
Diosgenin, dehydro	6o60	CUL7	−8.3
Deoxyvasicinone	4jt8	SIRT1	−8
Diosgenin acetate	6gu7	CDK1	−7.9
Deoxyvasicinone	7kkq	PARP1	−7.7
Diosgenin acetate	7kkq	PARP1	−7.7
Diosgenin, dehydro	5ij8	EZH2	−7.6
Diosgenin acetate	5ij8	EZH2	−7.5
Diosgenin, dehydro	7kkq	PARP1	−7.4
Diosgenin, dehydro	6g54	MAPK1	−7.3
Deoxyvasicinone	6gu7	CDK1	−7.2
Deoxyvasicinone	1b39	CDK2	−7.2
Deoxyvasicinone	5ij8	EZH2	−7.2
Deoxyvasicinone	4u6r	RACK1	−7
Diosgenin, dehydro	5wr7	NTRK1	−6.9
Diosgenin acetate	5wr7	NTRK1	−6.7
7-Epitaxol	6g54	MAPK1	−6.4
Deoxyvasicinone	6g54	MAPK1	−6.4
Deoxyvasicinone	6o60	CUL7	−6.3
Diosgenin acetate	6g54	MAPK1	−6.3
7-Epitaxol	4jt8	SIRT1	−5.7
7-Epitaxol	5ij8	EZH2	−5

## Data Availability

The raw data used to support the findings of this study are included within the article.

## Supplementary Materials

Supplementary Materials Supplementary Figure 1. HPLCs. (A) Standard solution; (B) Rhizoma Dioscoreae Nipponicae sample solution. Supplementary Table 1. The primers used in the qRT-PCR analysis.
