# Supplementary material for: A Network Pharmacology Approach to Reveal the Underlying Mechanisms of Rhizoma Dioscoreae Nipponicae in the Treatment of Asthma
Source: Evid Based Complement Alternat Med. 2022 Mar 30;2022:4749613. doi: 10.1155/2022/4749613 (PMC8986377; doi:10.1155/2022/4749613)
Supplement: Supplementary Materials — Supplementary Figure 1. HPLCs. (A) Standard solution; (B) Rhizoma Dioscoreae Nipponicae sample solution. Supplementary Table 1. The primers used in the qRT-PCR analysis. [file 4749613.f1.zip › 4749613.f1/Supplementary Table 1.docx]

Supplementary Table 1. The primers used in qRT-PCR analysis

| Gene | Primers sequences (5’- -3’) |
| --- | --- |
| PI3K | F: CGAGAGTGTCGTCACAGTGTC |
|  | R: TGTTCGCTTCCACAAACACAG |
| SIRT1 | F: TGATTGGCACCGATCCTCG |
|  | R: CCACAGCGTCATATCATCCAG |
| RACK1 | F: GTCTGCAAGTACACGGTCCA |
|  | R: AACAGAGTCTGGCCATCAGC |
| AKT | F: CCCTGCTCCTAGTCCACCA |
|  | R: TGTCTCTGTTTCAGTGGGCTC |
| CDK2 | F: GCGACCTCCTCCCAATATCG |
|  | R: GTCTGATCTCTTTCCCCAACTCT |
| NTRK1 | F: CAGTCTGATGACTTCGTTGATGC |
|  | R: CTCTTCACGATGGTTAGGCTTC |
| MAPK1 | F: TCAGATGAATTTTCGTTGGCAGA |
|  | R: AGCTTTTGTATTGGTCACAGCA |
| GAPDH | F: TGTGGGCATCAATGGATTTGG |
|  | R: ACACCATGTATTCCGGGTCAAT |
