# Supplementary figures and images for: A Network Pharmacology Approach to Reveal the Underlying Mechanisms of Rhizoma Dioscoreae Nipponicae in the Treatment of Asthma
Source: Evid Based Complement Alternat Med. 2022 Mar 30;2022:4749613. doi: 10.1155/2022/4749613 (PMC8986377; doi:10.1155/2022/4749613)

A

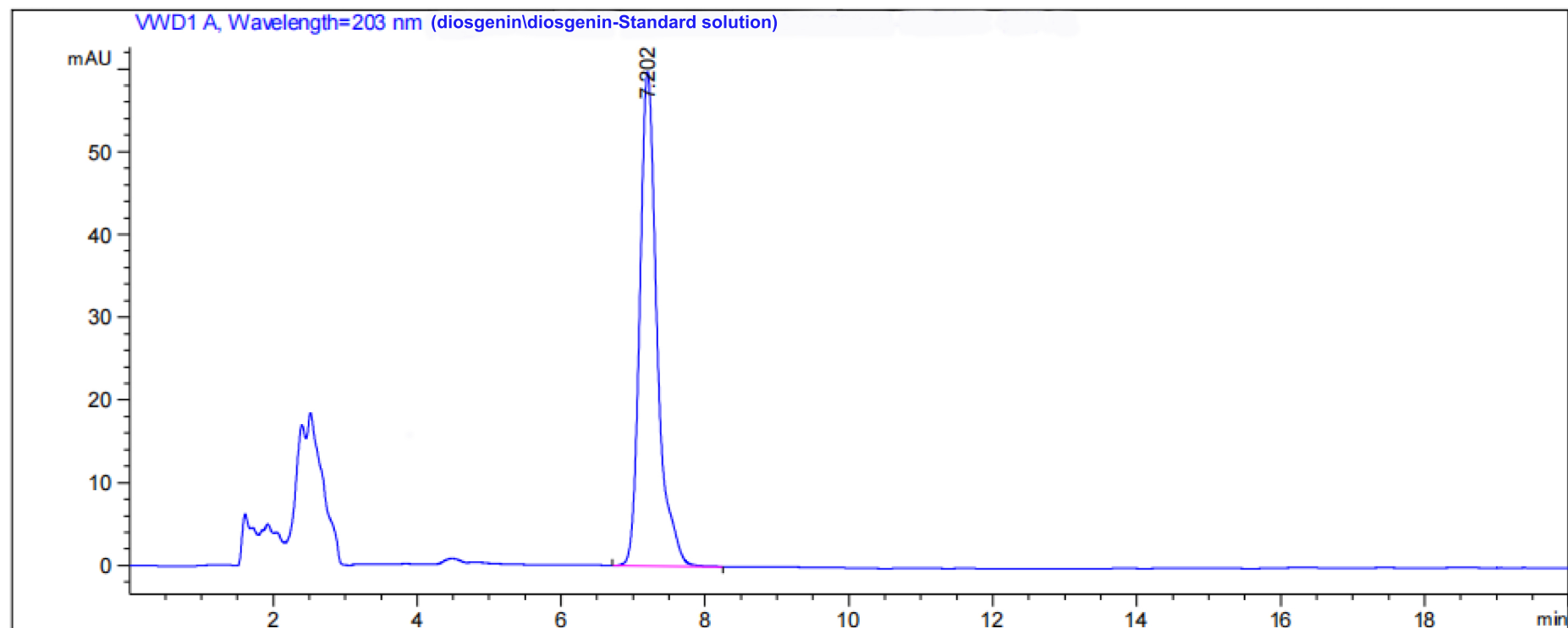

B

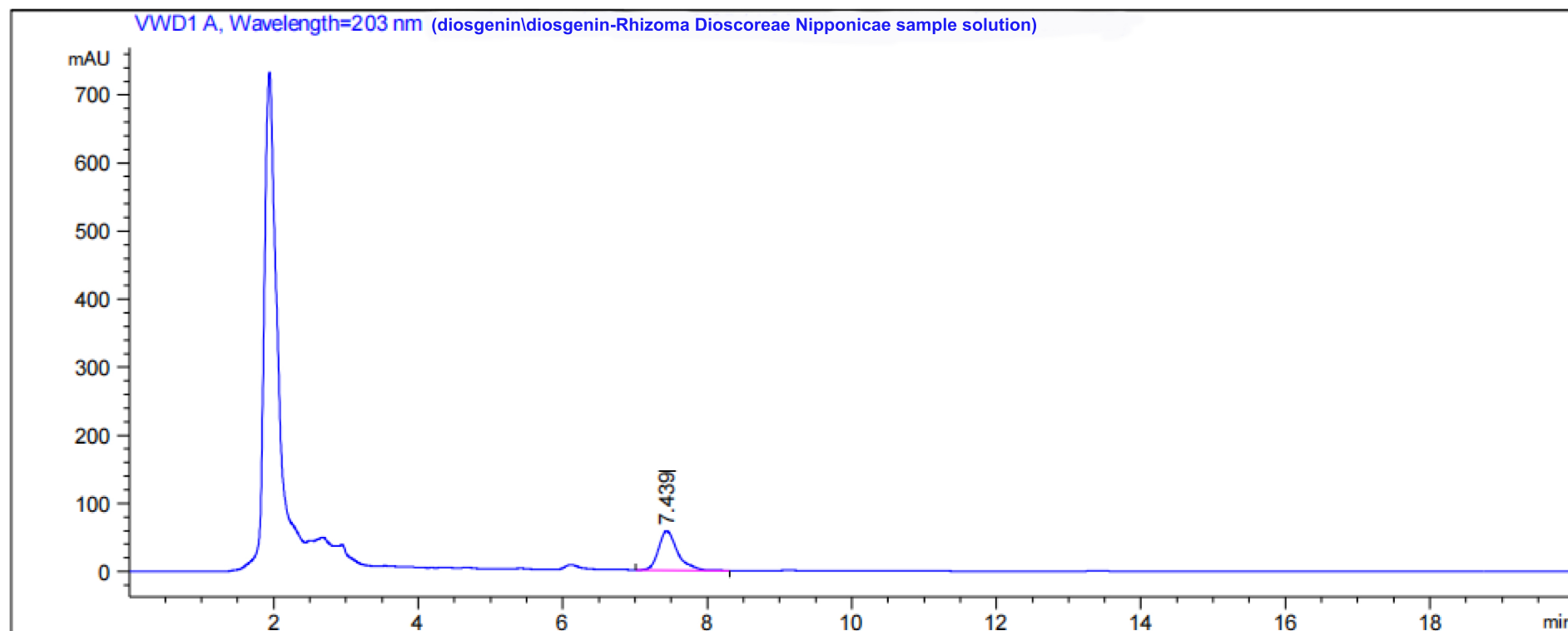

Supplement: Supplementary Materials — Supplementary Figure 1. HPLCs. (A) Standard solution; (B) Rhizoma Dioscoreae Nipponicae sample solution. Supplementary Table 1. The primers used in the qRT-PCR analysis. [file 4749613.f1.zip › 4749613.f1/Supplementary Figure 1 (1).pdf]
